# ROCK1/MLC2 inhibition induces decay of viral mRNA in BPXV infected cells

**DOI:** 10.1038/s41598-022-21610-9

**Published:** 2022-10-24

**Authors:** Ram Kumar, Yogesh Chander, Nitin Khandelwal, Assim Verma, Krishan Dutt Rawat, Brij N. Shringi, Yash Pal, Bhupendra N. Tripathi, Sanjay Barua, Naveen Kumar

**Affiliations:** 1 Present Address: National Centre for Veterinary Type Cultures, ICAR-National Research Centre on Equines, Hisar, India; 2 Department of Veterinary Microbiology and Biotechnology, Rajasthan University of Veterinary and Animal Sciences, Bikaner, India; 3 Present Address: Animal Science Division, Indian Council of Agricultural Research, Krishi Bhawan, New Delhi, India; 4 Department of Bio and Nano Technology, Guru Jambheshwar University of Science and Technology, Hisar, Haryana India

**Keywords:** Microbiology, Virology

## Abstract

Rho-associated coiled-coil containing protein kinase 1 (ROCK1) intracellular cell signaling pathway regulates cell morphology, polarity, and cytoskeletal remodeling. We observed the activation of ROCK1/myosin light chain (MLC2) signaling pathway in buffalopox virus (BPXV) infected Vero cells. ROCK1 depletion by siRNA and specific small molecule chemical inhibitors (Thiazovivin and Y27632) resulted in a reduced BPXV replication*,* as evidenced by reductions in viral mRNA/protein synthesis, genome copy numbers and progeny virus particles. Further, we demonstrated that ROCK1 inhibition promotes deadenylation of viral mRNA (mRNA decay), mediated via inhibiting interaction with PABP [(poly(A)-binding protein] and enhancing the expression of CCR4-NOT (a multi-protein complex that plays an important role in deadenylation of mRNA). In addition, ROCK1/MLC2 mediated cell contraction, and perinuclear accumulation of p-MLC2 was shown to positively correlate with viral mRNA/protein synthesis. Finally, it was demonstrated that the long-term sequential passage (P = 50) of BPXV in the presence of Thiazovivin does not select for any drug-resistant virus variants. In conclusion, ROCK1/MLC2 cell signaling pathway facilitates BPXV replication by preventing viral mRNA decay and that the inhibitors targeting this pathway may have novel therapeutic effects against buffalopox.

## Introduction

Buffalopox virus (BPXV) is a close variant of the vaccinia virus (VACV), the type-species of the genus *Orthopoxvirus*. BPXV causes pock-like lesions, primarily in domestic buffaloes (*Bubalus bubalis*) but cattle and humans can also be infected and hence, BPXV is considered as a potential zoonotic threat^1^. BPXV infection is primarily restricted to the Indian subcontinent but Egypt, Russia, and Italy have also experienced its infection^1–4^.

Buffalopox virus has a linear, dsDNA genome of about 195 kb in size^5^. Based on their time of expression, poxvirus genes are grouped early, intermediate and late. The early gene transcription occurs within the viral particles which allow the synthesis of mRNA soon after entry into the cytoplasm whereas intermediate and late transcription requires de novo viral protein synthesis and genome replication in cytoplasmic viral factories^6,7^. The cytoskeleton remodeling or motility is one of the crucial factors that add to the early phase of viral replication. It contributes to the perinuclear accumulation of the viral DNA at replication sites^6,7^, thereby assisting in viral entry, transcription/translation activities and genomic DNA replication^6^.

Currently there is no specific vaccine or antiviral drug available against BPXV infection. Most of the currently licensed antiviral drugs are based on directly disrupting the functions of viral proteins^8^. Due to the high rate of mutations in the viral genome, long-term selection pressure leads to selection of the viral mutants that become resistant at the druggable sites^9^. Kinases and phosphatase are implicated in various physiological processes to maintain cellular homeostasis. They have been extensively studied for their diverse role in cancer and small molecule chemical inhibitors (anticancer agents) have been developed against most of the kinases known so far. Besides cancer, kinome has also been implicated in virus replication and could be targeted for the development of antiviral drugs^10–12^. Rho-associated coiled-coil containing protein kinase (ROCK) belongs to the Rho subfamily, possess GTPase activity, and has been active as a molecular switch. There are two homologous isomers of ROCK in the cell: ROCK1 (ROCK-I, ROKβ or p160 ROCK) and ROCK2 (ROCK-II, ROKα or Rho-kinase). Rho/ROCK cell signaling pathway regulates cell morphology, polarity, and cytoskeletal remodeling by regulating phosphorylation of a wide variety of downstream substrates such as MLC2, LIM (Lin-11 Isl-1 Mec-3) Kinase (LIMK), Ezrin/Radixin/Moesin (ERM) and intermediate filament proteins^13^. Besides, activated ROCK signalling is also known to suppress cell proliferation and induce apoptosis by negative regulation of Akt/mTORC and ERK^14^. Many studies have shown that ROCK proteins play an important role in the genesis, survival, invasion and metastasis of tumor cells^15^. Viral infections have also been shown to induce the rearrangement of cell cytoskeleton and polarity^16–23^. The emerging evidences have established a link between virus infection and ROCK cell signalling pathway^24–28^. Depending on the nature of the virus, ROCK signaling may either promote or inhibit virus replication^24–28^. However, there is a significant gap in our understanding about the precise role of ROCK signaling in virus replication, particularly poxvirus infection. We screened some small molecule chemical inhibitors (commercially available) and identified Thiazovivin (a selective inhibitor of ROCK) as one of the inhibitors that reduced BPXV replication. Further studies demonstrated novel roles of ROCK1 signaling in BPXV replication viz; induction of cell contraction (that helps the virus to anchor at the replication sites) and in preventing viral mRNA decay.

## Results

### ROCK1 supports BPXV replication

**Figure 1:**
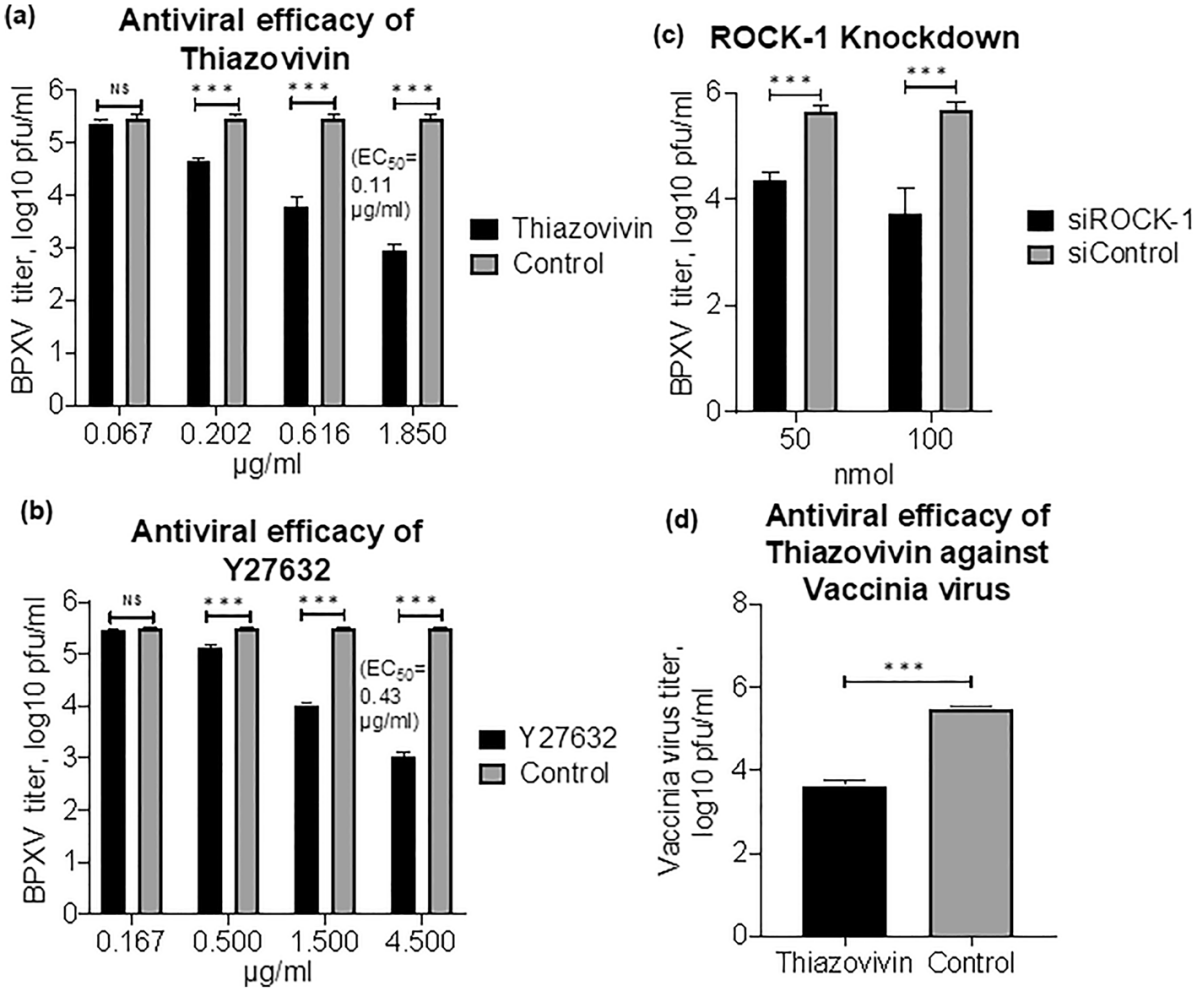
In vitro antiviral efficacy of ROCK1 inhibitor. (**a**) In vitro antiviral efficacy of Thiazovivin. Vero cells, in triplicates, were infected with BPXV at MOI of 0.1 in the presence of indicated concentrations of Thiazovivin or DMSO. The virus particles released in the cell culture supernatants at 48 hpi were quantified by plaque assay. n = 3 independent experiments. (**b**) In vitro antiviral efficacy of Y27632. Vero cells, in triplicates, were infected with BPXV at MOI of 0.1 in the presence of indicated concentrations of Y27632 or DMSO. The virus particles released in the cell culture supernatants at 48 hpi were quantified by plaque assay. n = 3 independent experiments. (**c**) siRNA knockdown. Vero cells, in triplicates, were transfected with ROCK1 (50 nmol and 100 nmol) and negative control (100 nmol) siRNAs, followed by BPXV infection at MOI of 1. The virus yields in the infected cell culture supernatants at 48 hpi were quantified by plaque assay. n = 3 independent experiments. (**d**) In vitro antiviral efficacy of Thiazovivin against vaccinia virus. Vero cells, in triplicates, were infected with vaccinia virus at MOI of 0.1 in the presence of Thiazovivin or DMSO. The virus particles released in the cell culture supernatants at 48 hpi were quantified by plaque assay. n = 3 independent experiments. CC_50_ and EC_50_ were determined by the Reed-Muench method. Error bars indicate SD. Pair-wise statistical comparisons were performed using Student’s t test (*NS* non-significant difference, ***P < 0.001).

In order to evaluate the effect of ROCK1 on BPXV replication, we used ROCK1-specific small molecule chemical inhibitors-Thiazovivin and Y27632. At non-cytotoxic concentrations (Supplementary Fig. 1a,b), both Thiazovivin and Y27632 inhibited BPXV replication in a dose-dependent manner (Fig. 1a,b). The effective concentration 50 (EC_50_) was determined to be 0.11 µg/ml and 0.43 µg/ml respectively for Thiazovivin and Y27632. The yield of infectious BPXV was also significantly low in the cells that received siRNA directed against ROCK1 as compared to the cells that received the negative control siRNA (Supplementary Fig. 2a,b; Fig. 1c). To analyze the virucidal effects on extracellular virions, BPXV was incubated with indicated concentrations of the inhibitors (Thiazovivin/Y27632) or DMSO for 1.5 h and then residual infectivity was titrated on Vero cells. Infectious viral titers were comparable in both inhibitor-and vehicle control-treated cells (Supplementary Fig. 3a,b), suggesting that ROCK1 inhibitors do not exert any direct virucidal effect on BPXV and that the antiviral activity of Thiazovivin/Y27632 is presumably due to the inhibition of viral life cycle in the target cells. Taken together, it was concluded that ROCK1signaling supports BPXV replication. Thiazovivin was also shown to suppress the vaccinia virus (VACV) yield in Vero cells (Fig. 1d), thereby suggesting that the ROCK1 requirement is conserved among orthopoxviruses.

### Thiazovivin suppresses post-entry steps of BPXV replication cycle

In order to examine which specific step(s) of BPXV life cycle could be affected by ROCK1 inhibitor (Thiazovivin), initially a time-of-addition assay was performed in the setting of one-step growth curve. Vero cells were infected with BPXV and the Thiazovivin- or vehicle-controls were applied at a timely interval from 1 to 36 hpi. The yield of infectious virus in the infected cell culture supernatant was quantified when one full cycle of the BPXV was likely to be completed, i.e. at 36–48 hpi^29^. The application of Thiazovivin resulted in almost similar levels of BPXV inhibition, either applied before infection (pre-treatment) or at 1 hpi and 6 hpi (Supplementary Fig. 4), suggesting that Thiazovivin does not inhibit the early step of BPXV life cycle (i.e. entry and attachment). The addition of inhibitor at later time points exhibited very low (18 hpi, 24 hpi, 30 hpi) or no inhibition (36 hpi), suggesting that Thiazovivin has no significant effect on the late stages of the BPXV life cycle (i.e. assembly/release of viruses). However, addition of Thiazovivin between 6 hpi to 18 hpi resulted in a significant reduction in the virus yield, thereby suggesting that Thiazovivin may target the post-entry stages of BPXV life cycle.

In order to determine the effect of Thiazovivin on the attachment of the BPXV to host cells, virus infection was carried out at 4 °C; it allowed attachment of the virus to the host cells but restricted viral entry. As indicated in Supplementary Fig. 5a, viral titres were comparable in both vehicle-control-treated and Thiazovovin-treated cells, implying that Thiazovivin does not affect BPXV attachment to the host cells. To evaluate the effect of Thiazovivin upon BPXV entry, the virus was first allowed to attach at 4 °C in the absence of inhibitor, followed by incubating of the cells at 37 °C for 1 h (it allowed viral entry) in presence of the inhibitor. Viral titres after completion of one full cycle viz; at 40 hpi, were comparable in both vehicle-control-treated and Thiazovivin-treated cells (Supplementary Fig. 5b), suggesting that ROCK1 inhibitor does not affect BPXV entry into the target cells. In the virus release assay, the inhibitor was applied at the time when early (attachment/entry) and middle stages (genome and protein synthesis) of the viral life cycle had occurred and when virus presumably started to release from the infected cells viz; at 36 hpi. Viral titres were comparable in both vehicle-control-treated and Thiazovivin-treated cells (Supplementary Fig. 5c), suggesting that ROCK1 inhibitor does not affect BPXV release from the infected cells.

### ROCK1 inhibition suppresses levels of viral mRNA, DNA and proteins

In order to determine the effect of Thiazovivin on BPXV DNA/mRNA synthesis, we initially determined the kinetics of the synthesis of BPXV DNA/mRNA wherein the Vero cells were infected at high MOI and the cell lysates were collected at various times post-infection to quantify the viral mRNA [cDNA of membrane protein (M gene)] by qRT-PCR. The levels of *M* gene was progressively increased from 8 to 12 hpi before starting declining at 16 hpi (Supplementary Fig. 6a). In contrast, there was a progressive increase in viral DNA synthesis from 12 to 24 hpi (Supplementary Fig. 6b).

**Figure 2:**
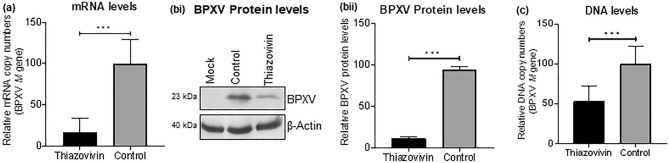
Effect of Thiazovivin on levels of BPXV mRNA/protein/DNA. (**a**) Viral mRNA. Confluent monolayers of Vero cells, in triplicates, were infected with BPXV (MOI of 5), followed by washing with PBS and addition of fresh medium. Inhibitor or vehicle-controls were applied at 4 hpi. Cells were scraped at 12 hpi to isolate RNA. cDNA was synthesized using oligo (dT) primers and quantified for BPXV *M* gene by qRT-PCR. Ct values were normalized with the β-actin housekeeping control gene and relative fold change was calculated by the ∆∆ Ct method. n = 3 independent experiments. (**b**) Viral proteins. Cells were infected with BPXV and treated with Thiazovivin as described above. The cell lysates were prepared at 24 hpi. The levels of viral (upper panel) and β-Actin house-keeping control protein (lower panel) were determined by Western blot analysis (bi). The histogram (bii) shows the band intensity of the protein. The blots were quantified by densitometry (ImageJ) and the data are presented as mean with SD. n = 3 independent experiments. See Supplementary Fig. 6a for full blots. (**c**) Viral DNA. Vero cells, in triplicates, were infected with BPXV and treated with Thiazovivin as described above. BPXV *M* gene in Thiazovivin-treated and vehicle-control-treated Vero cells were quantified by qRT-PCR. Ct values were normalized with the β-actin housekeeping control gene and relative fold change was calculated by the ∆∆ Ct method. n = 3 independent experiments. Error bars indicate SD. Pair-wise statistical comparisons were performed using Student's t-test (***P < 0.001; **P < 0.01).

To evaluate the effect of Thiazovivin on viral DNA/mRNA/protein synthesis, Vero cells were infected with BPXV at MOI of 5 and the inhibitor was applied at 4 hpi, a time point when early steps of the viral replication cycles (attachment/entry) were expected to occur. In order to evaluate the effect on mRNA, cells were scrapped at 12 hpi to isolate the RNA and quantify *M (*BPXV) and *β-actin* (housing keeping control) genes. As compared to the vehicle-control-treated cells, inhibitor-treated cells showed ~ 80% reduction in viral mRNA (Fig. 2a) suggesting that ROCK1 signalling may support the synthesis of viral mRNA. Since reduced viral mRNA synthesis may eventually reflect a reduction in synthesis of the viral proteins, levels of viral protein in inhibitor-treated cells were also low (Fig. 2bi–ii). Likewise, as compared to the vehicle-control-treated cells, inhibitor-treated cells showed moderate (~ 50%) reduction in viral DNA (Fig. 2c). Taken together, it was concluded that Thiazovivin reduces levels of viral DNA/mRNA.

### ROCK1 inhibition promotes deadenylation/decay of BPXV mRNA

**Figure 3:**
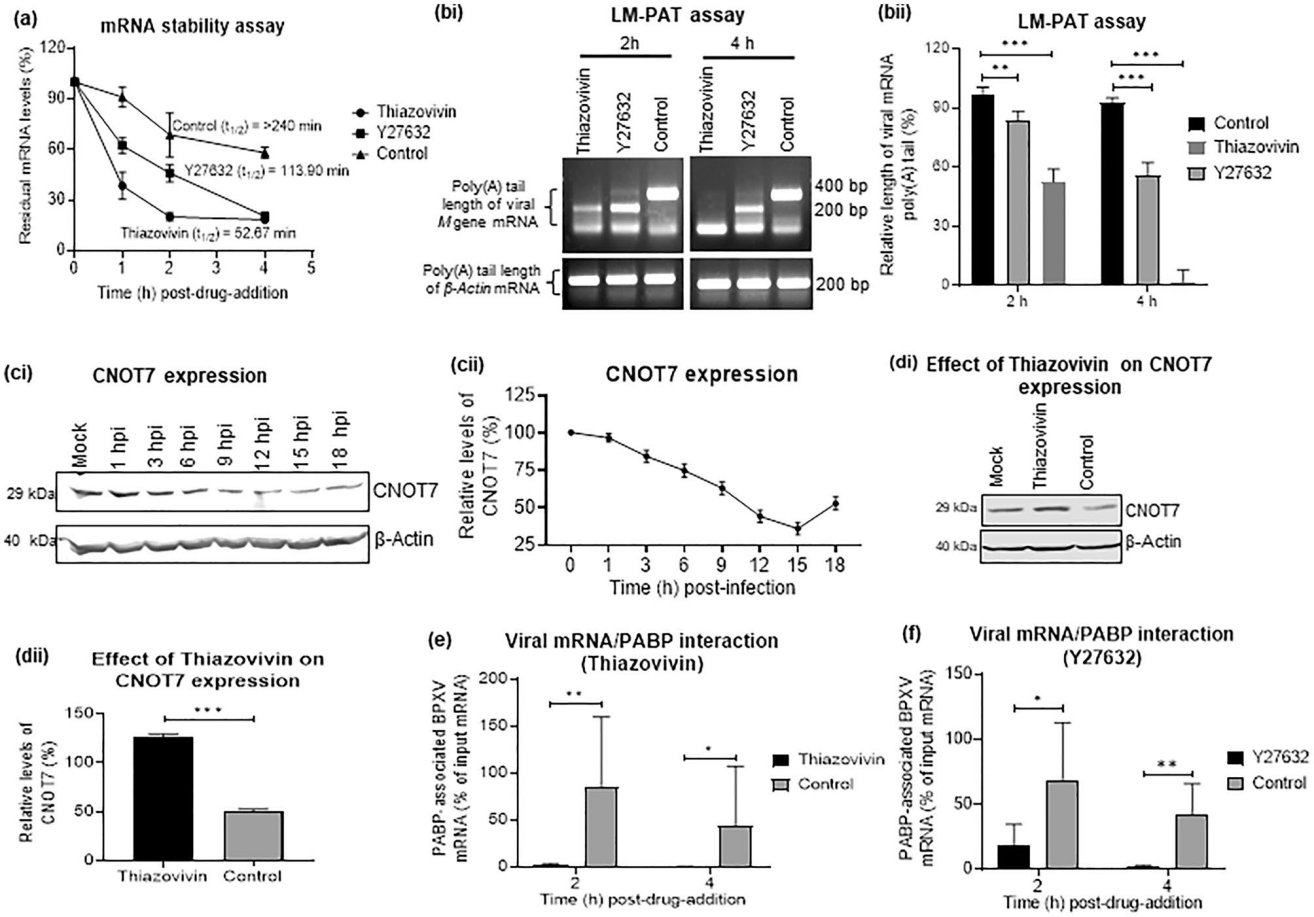
Thiazovivin induces viral mRNA decay. (**a**) Measurement of mRNA stability: Vero cells, in triplicates, were infected with BPXV at MOI of 5. At 9 hpi, cells were treated with Thiazovivin (1 µg/ml), Y27632 (1.5 µg/ml) or equivalent volumes of DMSO in the presence of Actinomycin D (5 µg/ml). Cells were subjected to RNA isolation at indicated time points (post-drug treatment) and subjected to cDNA synthesis and quantified for BPXV *M* gene by qRT–PCR. The relative levels of viral mRNA at 0 h, 1 h, 2 h and 4 h in Thiazovivin or Y27632- and control-treated cells are shown. n = 3 independent experiments. (**b**) LM-PAT assay. Vero cells were infected with BPXV. At 9 hpi, the cells were treated with the inhibitors (Thiazovivin or Y27632) or DMSO in the presence of Actinomycin D. At indicated times post-drug-addition, cells were scrapped to isolate the RNA. The RNA was allowed to react with an adaptor oligo(dT) primer in the presence of T4 DNA ligase and subjected to cDNA synthesis. The length of poly(A) tail of viral (*M*) and cellular (β-actin) mRNA were determined by PCR by using an anchor primer and a gene-specific primer (bi). The length of the PCR product (BPXV *M* gene) in inhibitor-treated and control-treated cells was measured (ImageJ) and the relative length of the viral mRNA poly(A) tail (% of control) at different times treatment was calculated (bii). Data are presented as mean with SD. n = 3 independent experiments. See Supplementary Fig. 6b for full gel. (**c**) Kinetics of CNOT7 expression. Vero cells were either mock-infected or infected with BPXV at MOI of 5. Cell lysate were prepared at indicated time points and subjected for detection of CNOT7 levels in Western blot analysis. The levels of CNOT7 (upper panel) and β-actin (house-keeping control protein, lower panel) are shown (ci). The line graph (cii) shows the band intensity of the protein at different times post-infection. The blots were quantified by densitometry (ImageJ) and the data are presented as mean with SD. n = 3 independent experiments. See Supplementary Fig. 6b for full blots. (**d**) Effect of Thiazovivin on CNOT7 expression. Vero cells were either mock-infected or infected with BPXV at MOI of 5. Thiazovivin or vehicle controls were added at 6 hpi. Cell lysates were prepared at 9 hpi and subjected for detection of CNOT7 levels in Western blot analysis (di). The histogram (dii) shows the band intensity of the protein. The blots were quantified by densitometry (ImageJ) and the data are presented as mean with SD. n = 3 independent experiments. See Supplementary Fig. 6c for full blots. (e and f) ROCK1 inhibition blocks interaction of BPXV mRNA with PABP (CHIP assay).Vero cells, in triplicates, were infected with BPXV at MOI of 5. At 9 hpi, the cells were treated with ROCK1 inhibitors (Thiazovivin and Y27632) or vehicle control. At 2 h and 4 h post-drug addition, the cell lysates were prepared as per the procedure described for CHIP assay (materials and method section). The clarified cell lysates were incubated with α-PABP or equivalent volume of the IP buffer (Beads control), followed by incubation with Protein A Sepharose® slurry. The beads were then washed five times in the IP buffer. To reverse the cross-linking, the complexes were then incubated with Proteinase K. Finally, the reaction mixtures were centrifuged and the supernatant was subjected to cDNA preparation and quantitation of BPXV RNA (M gene) by qRT-PCR. The percentage of the input BPXV mRNA bound to PABP in Thiazovivin-treated (**e**) and Y27632-treated cells (**f**) is shown. n = 3 independent experiments. Error bars indicate SD. Pair-wise statistical comparisons were performed using Student's t-test (***P < 0.001; **P < 0.01; *P < 0.05).

We investigated the effect of ROCK1 inhibition on the stability of viral mRNA. When the infected cells are expected to accumulate significant amount of viral RNA (~ 9 hpi), cells were treated with Thiazovivin/Y27632- or DMSO in the presence of Actinomycin D and followed by quantitation of viral mRNA at 0 h, 1 h, 2 h and 4 h post drug-treatment). The relative levels of BPXV mRNA was significantly low in Thiazovivin and Y27632-treated cells (t_1/2_ = 52.67 min and t_1/2_ = 113.50 min respectively) as compared to the vehicle control-treated cells (t_1/2_ =  > 240 min) (Fig. 3a). This suggested that ROCK1 inhibition induces viral mRNA decay in the target cells.

Further, we evaluated the stability/intactness of poly(A) tail of viral mRNA by LM-PAT assay wherein, at 9 hpi, the BPXV infected cells were treated with Thiazovivin/Y27632- or DMSO in the presence of Actinomycin D. RNA isolated from Thiazovivin/Y27632 or DMSO-treated cells at 2 h and 4 h post-drug treatment was allowed to react with an adaptor oligo(dT) primer in the presence of T4 DNA ligase and subjected to cDNA synthesis. The length of poly(A) tail of viral (*M*) and cellular (*β-actin*) mRNA were determined by PCR amplification. As compared to the DMSO-treated cells, a significant reduction in poly(A) tail of BPXV mRNA (M gene) was observed in both Thiazovivin- and Y27632-treated cells whereas no effect was observed on the poly(A) tail length of endogenous mRNA (Fig. 3bi–ii). This suggested that ROCK-1 inhibition specifically induces deadenylation (decay) of viral mRNA.

### BPXV infection induces a progressive decrease in CNOT7 expression which can be reversed by Thiazovivin

We further explored the mechanisms by which ROCK1 inhibition leads to deadenylation of viral mRNA. The recruitment of cellular deadenylase complex CCR4-NOT (catalytic active subunit protein CNOT7 with 3′-5′ exonuclease activity) at poly (A) tail of mRNA induces the displacement of PABP (translational repression) which eventually promotes degradation of the mRNA and hence the blockade of the translation^30,31^. We observed that BPXV infection results in a progressive decrease in CNOT7 expression from its basal level, the lowest being at 9 to 18 hpi (Fig. 3ci–ii). Contrarily, Thiazovivin treatment resulted in the regain of the loss of CNOT7 expression (Fig. 3di–ii). These evidences appear to suggest the involvement of CCR4-NOT in ROCK1 inhibition-associated mRNA decay.

### Thiazovivin blocks interaction of BPXV mRNA with PABP

PABP is a poly (A)-binding protein which prevents degradation of mRNA without affecting protein synthesis^30,31^. In order to further confirm that reduced levels of viral mRNA in Thiazovivin-treated cells are due to decreased stability and not due to its effect on the synthesis of viral RNA, we performed a CHIP assay by using α-PABP antibody, wherein, a significant reduction (~ 70%) of PABP-associated viral mRNA was observed in Thiazovivin-treated (Fig. 3e) and Y27632-treated cells (Fig. 3f) as compared to the vehicle-control-treated cells. This suggested that Thiazovivin blocks interaction of BPXV mRNA with PABP which may eventually result in the repression of viral mRNA translation.

### BPXV infection induces ROCK1/p-MLC2-mediated cell contraction

**Figure 4:**
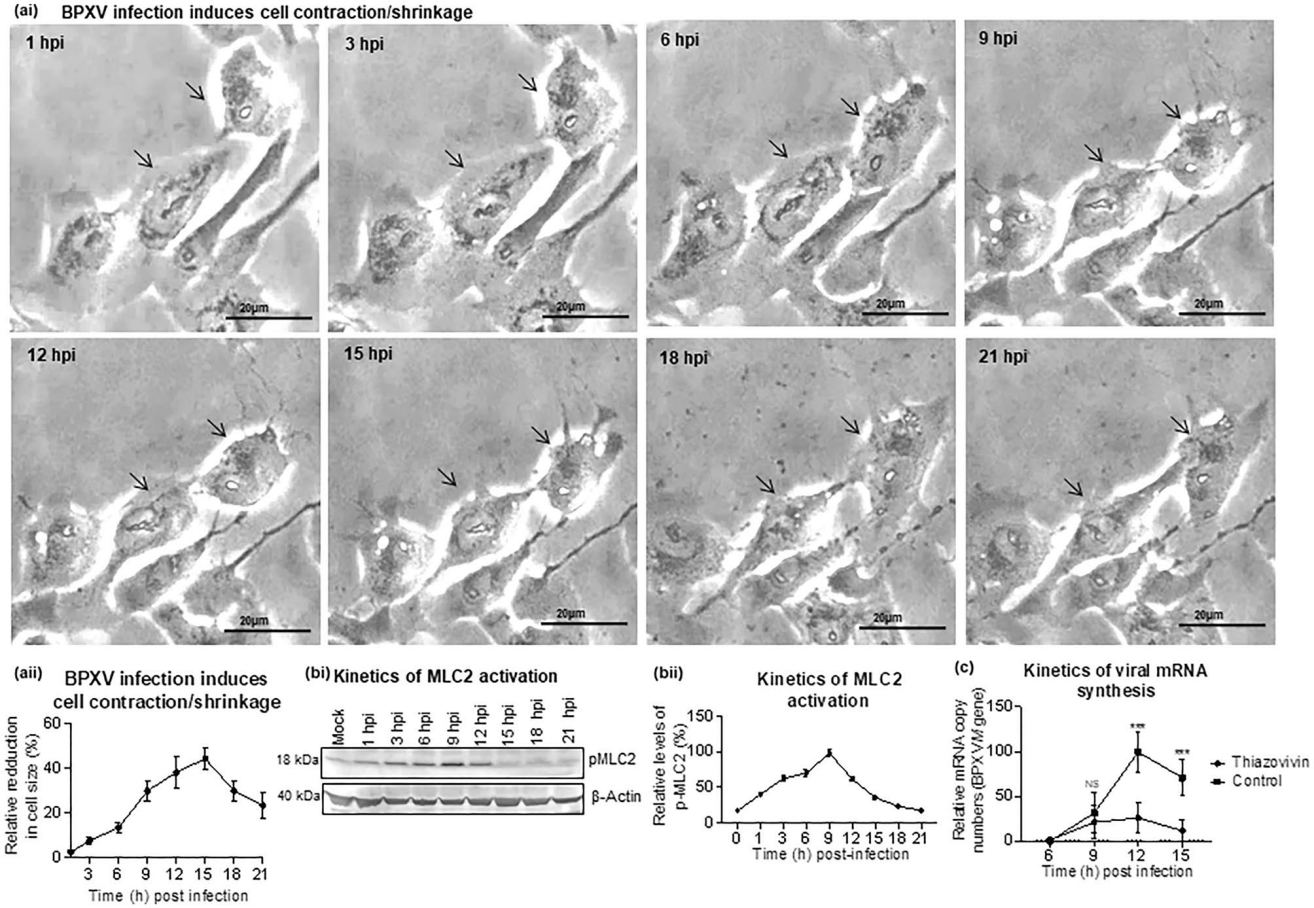
ROCK1/p-MLC2 signaling mediated cell contraction following BPXV infection is prerequisite for the stability of viral mRNA. (**a**) BPXV infection induces cell contraction (membrane blebbing). Vero cells were grown in chamber slides and infected with BPXV at an MOI of 5 for 1 h. Cells were subjected to live imaging. Cell morphology of BPXV infected cells at different time points are shown (ai). The histogram shows the relative reduction in cell size at different times post-BPXV infection (aii). To quantify BPXV-induced cell shrinkage, fifty cells were selected and the change in their size was measured by ImageJ at different times post-infection. The relative change in cell size (shrinkage) was expressed as a % of cell size immediately after viral infection (~ 1hpi) and the data are presented as mean with SD. (**b**) Kinetics of MLC2 activation. Vero cells were either mock-infected or infected with BPXV at MOI of 5. Cell lysates were prepared at indicated time points and subjected for detection of p-MLC2 levels in Western blot analysis. The levels of pMLC2 (upper panel) and β-actin (house-keeping control protein, lower panel) are shown (bi). The line diagram (bii) shows the band intensity of the protein. The blots were quantified by densitometry (ImageJ) and the data are presented as mean with SD. n = 3 independent experiments. See Supplementary Fig. 6d for full blots (**c**) Kinetics of viral mRNA synthesis in Thiozovivin-treated and vehicle control treated cells. Vero cells, in triplicates, were infected with BPXV at MOI of 5. Thiazovivin was applied at 3 hpi and cell lysate were prepared at the indicated time points for quantitation of viral mRNA (*M* gene). n = 3 independent experiments. Error bars indicate SD. Pair-wise statistical comparisons were performed using Student's t-test (***P < 0.001, *NS* non-significant).

During initial experiments, it was observed that BPXV-infected cells undergo membrane blebbing or rounding (contraction), most profoundly at 9–15 hpi (Fig. 4ai–ii; Supplementary Video 1). To provide further possible mechanistic insights, we evaluated the phosphorylation kinetics of MLC2 (a key ROCK1 substrate involved in cell contractibility and motility) in BPXV infected cells. BPXV infection was shown to induce MLC2 phosphorylation as early as 1hpi, with a peak expression at 9 hpi-12 hpi (Fig. 4bi–ii). Likewise, the viral mRNA levels were also peak at 9 hpi-15 hpi (Fig. 4c). In conclusion, cell contraction, MLC-2 phosphorylation and the levels of viral mRNA in BPXV infected cells correlated with each other.

**Figure 5:**
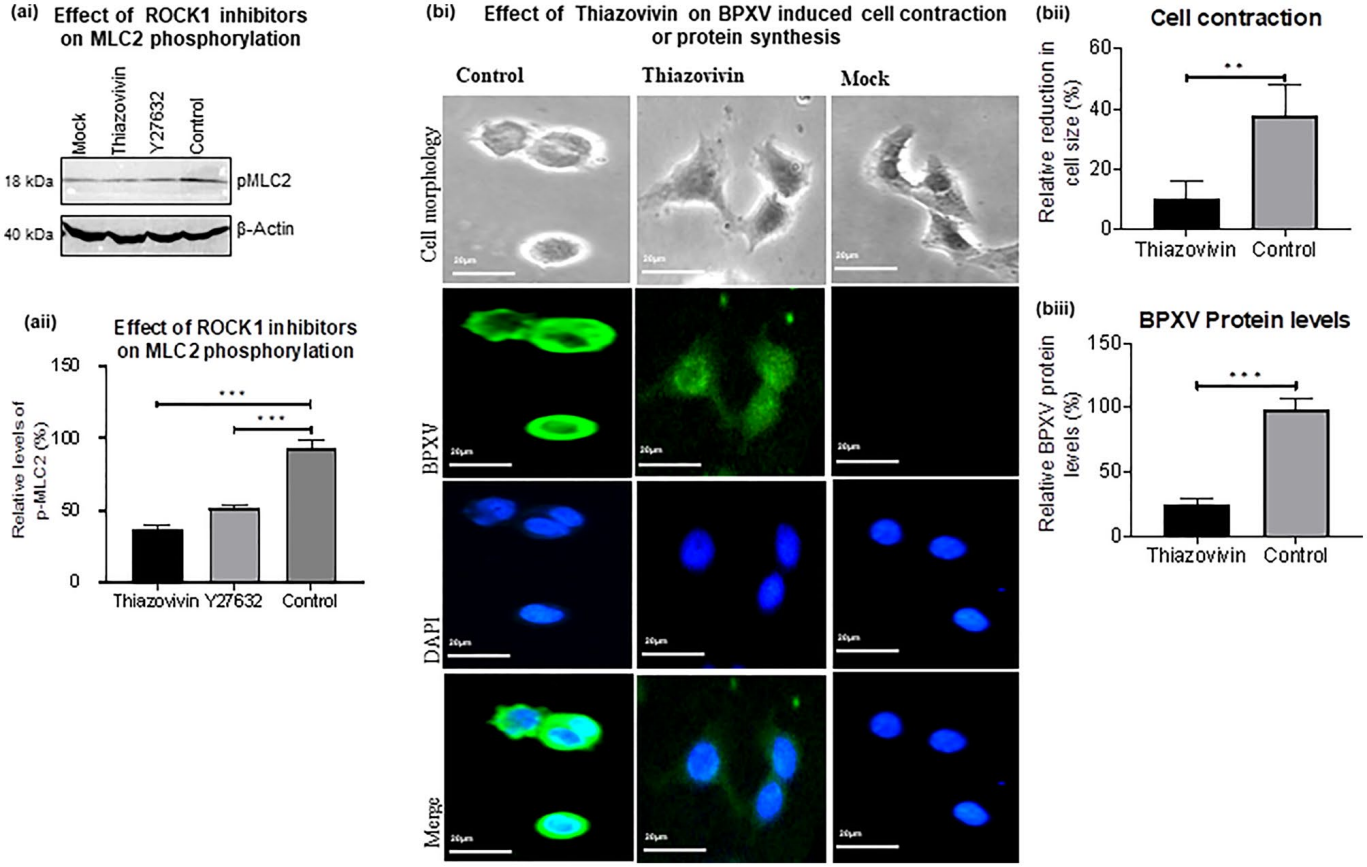
ROCK1 inhibition impairs BPXV-induced cell contraction and viral protein synthesis. (**a**) Effect of Thiazovivin on MLC2 phosphorylation (activation). Vero cells were either mock-infected or infected with BPXV at MOI of 5. Thiazovivin or vehicle controls were added at 4 hpi. Cell lysates were prepared at 9 hpi and subjected for detection of the p-MLC2 levels in Western blot analysis (ai). The histogram (aii) shows the band intensity of the protein. The blots were quantified by densitometry (ImageJ) and the data are presented as mean with SD. n = 3 independent experiments. See Supplementary Fig. 6e for full blots. (**b**) Effect of Thiazovivin on BPXV induced cell contraction and levels of viral proteins. HeLa cells were grown in chamber slides and infected with BPXV at an MOI of 5 for 1 h. Thiazovivin was applied at 4 hpi. At 15 hpi, BPXV (FITC) proteins were probed by immunofluorescence assay. Cell morphology and level of viral proteins of Thiazovivin-treated or untreated cells is shown (bi). The histogram shows the relative reduction in cell size (bii) and relative levels of BPXV proteins (biii) in Thiazovivin treated or untreated cells. The area (n = 50 cells) and the intensity of viral proteins (n = 50 cells) were quantified by ImageJ. The data are presented as mean with SD. Pair-wise statistical comparisons were performed using Student's t-test (***P < 0.001; **P < 0.001).

**Figure 6:**
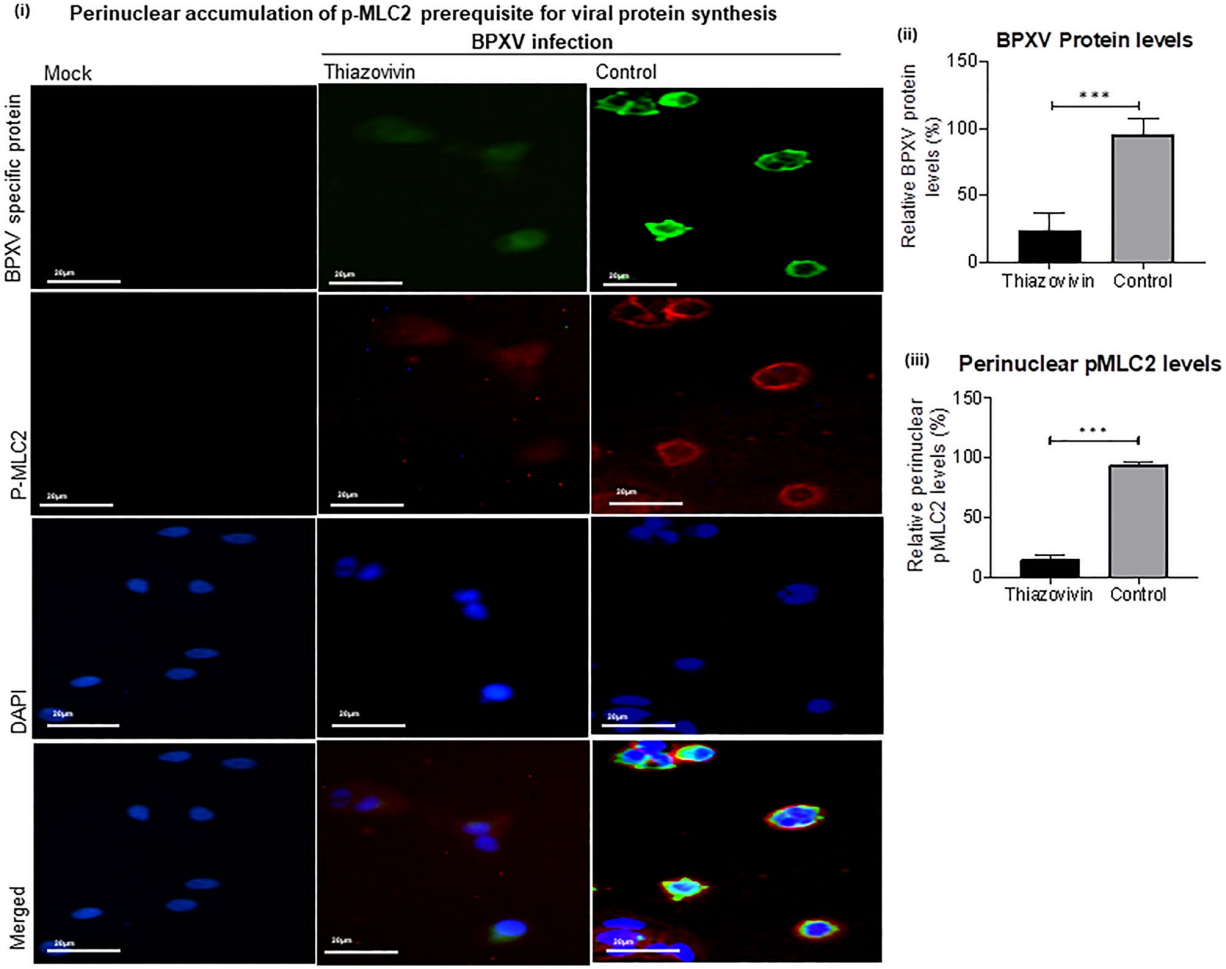
Perinuclear accumulation of p-MLC2 is prerequisite for the synthesis of viral proteins. HeLa cells were grown in chamber slides and infected with BPXV at an MOI of 5 for 1 h. Thiazovivin was applied at 4 hpi. At 15 hpi, BPXV (FITC) proteins and p-MLC2 (Rhodamine) were probed by immunofluorescence assay (i). The line graph shows the levels of BPXV protein (ii) and levels of perinuclear accumulation of p-MLC2 (iii) in BPXV infected cells. The intensities of viral proteins from fifty BPXV infected cells were quantified by densitometry (ImageJ) and the data are presented as mean with SD. Pair-wise statistical comparisons were performed using Student's t-test (***P < 0.001).

Thiazovivin treatment inhibited phosphorylation of MLC2 (Fig. 5ai–ii) and concomitantly the inhibition of the cell contraction (maintaining normal cell morphology, Supplementary Video 2) and BPXV protein synthesis (Fig. 5bi–iii). Furthermore, immunofluorescence assay indicated a correlation between perinuclear enrichment of p-MLC2 and the level of viral proteins in the infected cells (Fig. 6i–iii). The treatment of Thiazovivin impaired perinuclear enrichment of p-MLC2 which was in concomitant with reduced viral mRNA (Fig. 4c) and proteins in the target cells (Fig. 6i–iii). Taken together, it was concluded that BPXV induces ROCK1/p-MLC2-mediated cell contraction which might be essential for sequestration of the cellular factors that aid in mRNA stability and translation of the viral proteins.

**Figure 7:**
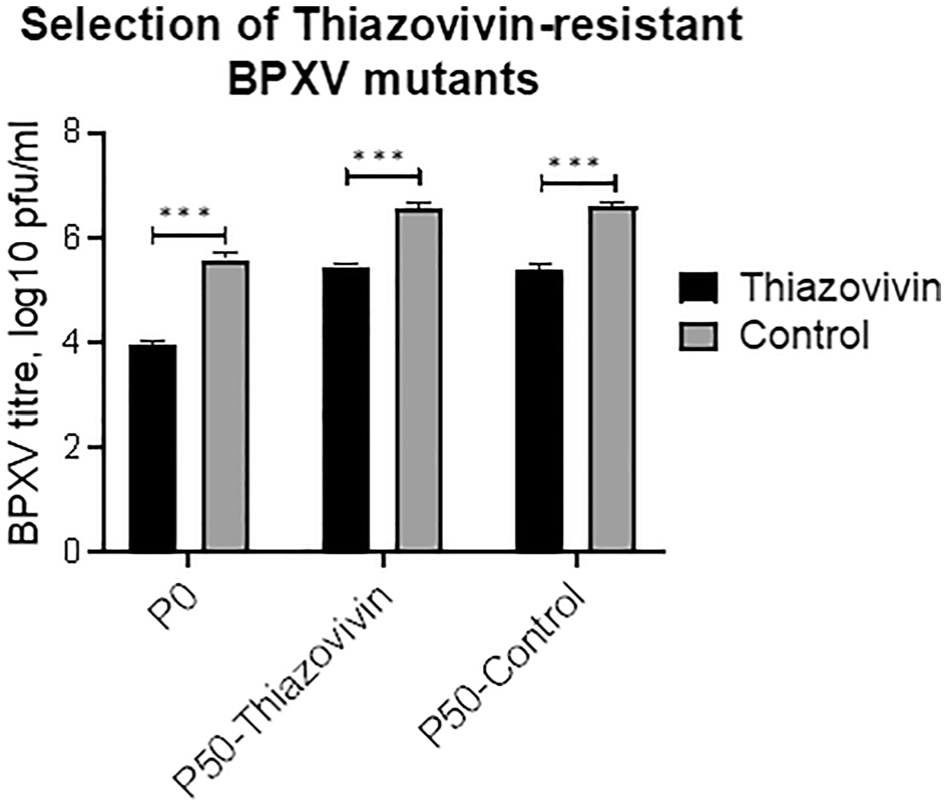
Evaluation of antiviral drug resistance against Thiazovivin. BPXV was sequentially passaged (P) fifty times in the medium containing 0.5 μg/ml of Thiazovivin or equivalent volumes of DMSO. The original virus stock (P0), P50-Thiazovivin and P50-Control viruses were used to infect Vero cells, in triplicates, at an MOI of 0.1 and treated with either 1 μg/ml of Thiazovivin or equivalent volumes of DMSO. The virus released in the supernatant at 48 hpi was quantified by plaque assay. Error bars indicate SD. Pair-wise statistical comparisons were performed using Student’s t test (***P < 0.001).

**Figure 8:**
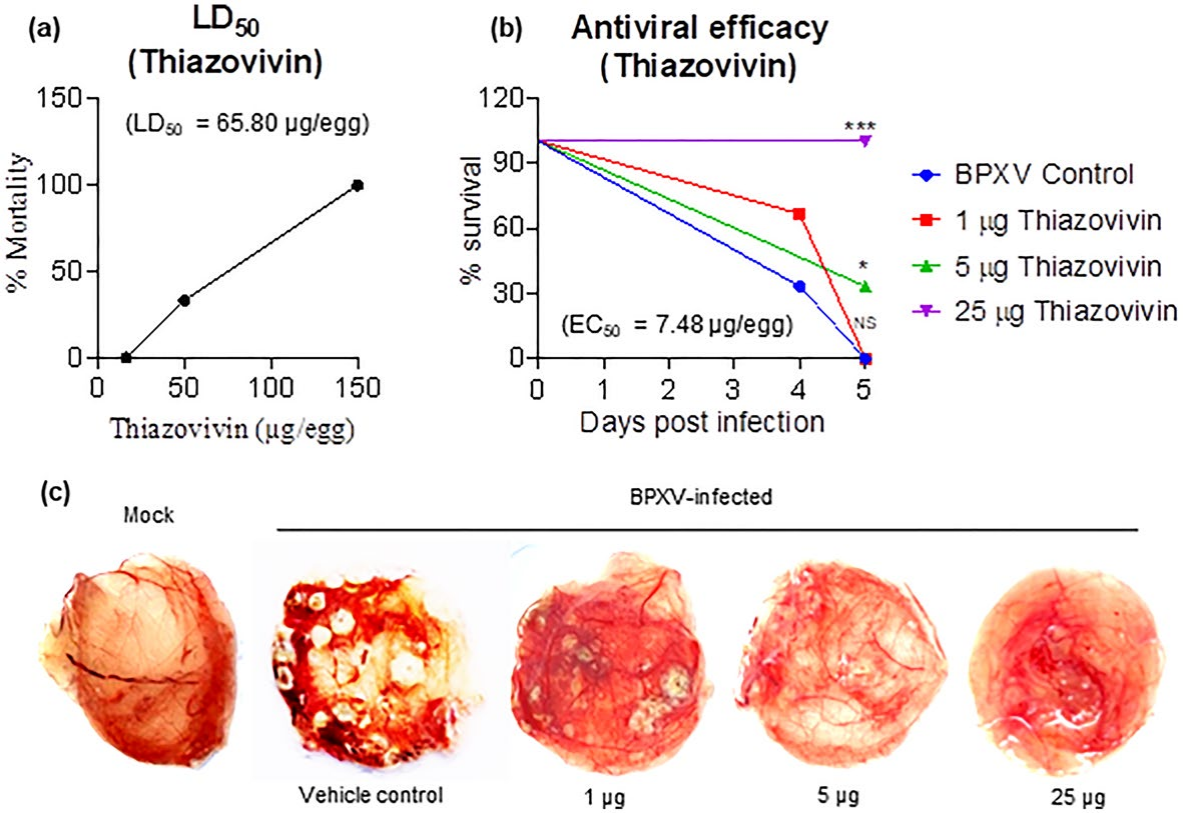
In *ovo* antiviral efficacy of Thiazovivin against BPXV. (**a**) *LD*_50_. Embryonated SPF chicken eggs, in triplicates, were inoculated with indicated concentration of Thiazovivin via CAM route. At 5 days post-Thiazovivin inoculation, eggs were visualized for viability of the embryos. LD_50_ was determined by the Reed-Muench method. (**b**) Anti-BPXV efficacy (EC_50_). Embryonated SPF chicken eggs, in triplicate, were inoculated with indicated concentration of Thiazovivin, followed by infection with BPXV at 100 EID_50_. At 5 days post-Thiazovivin inoculation, eggs were visualized for viability of the embryos (**c**) Pock lesions on CAM at various drug regimens are shown. n = 3 independent experiments. Pair-wise statistical comparisons were performed using Student’s t test (***P < 0.001; *P < 0.05, *NS* nonsignificant).

### Selection of Thiazovivin-resistant virus variants

In order to evaluate the development of drug-resistant virus variants, BPXV was serially passaged 50 times in the presence of Thiazovivin or vehicle-control (DMSO). At P50, Thiazovivin-passaged and control-passaged viruses were evaluated for their sensitivity to Thiazovivin. Like virus at P0, both P50-Thiazovivin and P50-control viruses exhibited equal susceptibility (~ 1.1 to 1.6 log difference in viral titer) to Thiazovivin (Fig. 7), suggesting that long-term passage in the presence of Thiazovivin does not induce the development of drug-resistant virus variants. However, as compared to the virus at P0, P50-Thiazovivin and P50-Control passaged viruses replicated much faster (~ 1 log higher titer) (Fig. 7) which could be due to increased fitness of the virus at higher passage levels.

### *In ovo* antiviral efficacy of Thiazovivin against BPXV

Members of the family *Poxviridae* can infect chicken embryos and cause distinctly visible lesions (pocks) on CAM of the embryonated chicken eggs^29,32^. We exploited this model to evaluate the *in ovo* efficacy of Thiazovivin against BPXV. Mortality of the embryo was observed at Thiazovivin concentration ≥ 50 μg/egg but not at 16.67 μg/egg (Fig. 8a). The LD_50_ was determined to be 65.80 μg/egg. For evaluation of anti-BPXV efficacy of Thiazovivin, eggs were infected with BPXV at 100 EID_50_ along with three different concentrations (25, 5, and 1 μg/egg) of Thiazovivin. Thiazovivn provided protection from BPXV-associated mortality in a dose-dependent manner (Fig. 8b). The EC_50_ was determined to be 7.48 μg/egg. As compared to the vehicle-control, no obvious pock lesions could be observed in Thiazovivin inoculated eggs (protected groups) (Fig. 8c). Taken together, it was concluded that Thiazovivin prevents the development of BPXV-induced pock lesions on CAM as well as the associated mortality.

## Discussion

Rho/ROCK cell signaling pathway regulates cell morphology, polarity, and cytoskeletal remodeling by regulating phosphorylation of a wide variety of downstream substrates^33^. Viral infections have also been shown to induce rearrangement of cell cytoskeleton and polarity^27^. The emerging evidences have established a link between virus infection and ROCK cell signaling pathway^24,26–28^, although the precise mechanism underlying regulation of virus replication by ROCK signaling remains elusive.

By regulating cell morphology, polarity, and cytoskeletal remodeling, Rho/ROCK signaling pathway induces several distinct changes in virus infected cells^34,35^, the most profound effect of which is enhancement of the virus yield^28,36–40^. In our study, ROCK1 inhibition (small molecule chemical inhibitors and siRNAs) resulted in reduced BPXV replication, as evident by reductions in the virus yield, genome copy numbers, viral mRNA/protein synthesis, without affecting other steps of viral life cycle such as attachment, entry and budding. This suggests that ROCK1 signaling is a prerequisite for BPXV replication.

Like vaccinia virus^41,42^, we observed that BPXV-infected cells undergo cell contraction (membrane blebbing/rounding) during early-to-middle stages of BPXV replication. Since viral assembly and egress of progeny virus particles require cessation of the cell motility, the infected cells are restored towards normal morphology during the late hours of infection^43^. Interestingly, in this study, we observed that the addition of Thiazovivin restores the morphology of cells which otherwise undergo contraction in BPXV infected. We further demonstrated that ROCK1-associated cell contraction in BPXV-infected cells is mediated via activation (phosphorylation) of MLC2. Also, there appears to be a correlation between cell contraction/perinuclear accumulation of p-MLC2 and synthesis of viral RNA/protein. Most importantly, ROCK1 was not shown to directly affect the synthesis of viral mRNA but rather prevents its degradation, a process which was shown to be mediated via inhibiting recruitment of host deadenylase complex (CCR4-NOT) and displacement of PABP. This is a novel role of ROCK1 signaling we have explored in the viral life cycle. It appears that BPXV induced cell contractions and perinuclear accumulation of p-MLC2 are essential for the sequestration of cellular factors that participate in regulating mRNA stability and translation of the viral proteins^7^.

Thiazovivin also provided significant therapeutic effect against lethal infection with BPXV in embryonated chicken eggs, suggesting its in vivo potential as an antiviral agent against poxvirus infections. Since Thiazovivin can inhibit cell metabolism, its long term use may eventually result in cytotoxicity^44–46^. Therefore, its further validation, in vivo efficacy and clinical trials are essential before actually introducing it from the research into the clinical settings.

Viruses rapidly develop drug-resistant variants, therefore, developing antiviral therapeutics is a major challenge^11,47,48^. Whereas direct virus-acting antiviral agents are well known to rapidly induce drug-resistant phenotype^11^, host-directed antiviral agents are less prone to induce the generation of drug-resistant phenotypes^49–55^. To evaluate the development of drug-resistant virus variants, BPXV was serially passaged 50 times in the presence of Thiazovivin or DMSO. Like virus at P0, both P50-Thiazovivin and P50-Control viruses showed equal susceptibility to Thiazovivin. However, as compared to the virus at P0, P50-Thiazovivin and P50-Control viruses replicated much faster (~ 1 log higher titer) and produced plaques that were of larger size. This could be due to the fitness advantages during long-term culture of the virus in cell culture.

## Conclusion

ROCK1 inhibition promotes deadenylation of viral mRNA (mRNA decay), mediated via inhibiting interaction of the viral mRNA with PABP and enhancing the expression of CCR4-NOT. BPXV infection induces cell contraction which is mediated via ROCK1/MLC2 cell signaling pathway. The cell contractions and perinuclear accumulation of p-MLC2 support viral protein synthesis. Therefore ROCK1/MLC-2 signaling may serve as a host-target for the development of novel antiviral therapeutics against poxvirus infections.

## Materials and methods

### Chemicals

ROCK1 inhibitor-Thiazovivin and Y27632 (Supplementary Fig. 7), MTT [3-(4,5-dimethyl-2-thiazolyl)-2,5-diphenyl-2H-tetrazolium bromide] and dimethyl sulfoxide (DMSO) were procured from Sigma (Steinheim, Germany).

### Cell culture

African green monkey kidney (Vero) and HeLa cells were received from National Centre for Cell Science (NCCS), Pune, India. Cells were grown in Dulbecco's Modified Eagle's Medium (DMEM) supplemented with 5–10% fetal calf serum (FBS) (Sigma, St. Louis, USA) and antibiotics.

### Virus

Vero cell adapted BPXV (Accession Number, VTCC-AVA90) was available at NCVTC Hisar. Vaccinia virus (VACV) was procured from American Type Culture Collection (ATCC). Both viruses were amplified and quantitated by plaque assay in Vero cells, as described previously^55^. The viral titers were determined as plaque forming units/ml (pfu/ml).

### Antibodies

ROCK1 Rabbit monoclonal antibody, p-MLC2 Mouse monoclonal antibody, CNOT7 Rabbit Monoclonal antibody, β-actin Mouse Monoclonal antibody, PABP1 Rabbit Monoclonal antibody and p44/42 MAPK (ERK1) Rabbit Monoclonal antibody were procured from Cell Signalling Technology (Massachusetts, USA). Hyperimmune serum that reacts with 23 kDa and 37 kDa BPXV proteins was raised in rabbits and has been previously described by our group^55^.

### Cytotoxicity and virucidal activity

The cytotoxic^55^ and virucidal^11^ effects of Thiazovivin (Supplementary Fig. 1a) and Y27632 (Supplementary Fig. 1b) were determined as previously described.

### In vitro antiviral efficacy of Thiazovivin (EC_50_)

Vero cells, in triplicates were infected with BPXV at MOI of 0.1 for 1 h followed by washing with PBS, and addition of fresh DMEM containing either DMSO or three-fold serial dilutions of Thiazovivin (50 µg/ml to 0.002 µg/ml). Viral titers in the infected cell culture supernatants at 48 hpi were determined by plaque assay. Effective concentration 50 (EC_50_), concentration of the inhibitor required to reduce 50% virus yield) was determined by the Reed-Muench method.

### Time-of-addition assay

Confluent monolayers of Vero cells, in triplicates were infected with BPXV at MOI of 5, followed by addition of Thiazovivin (1 µg/ml) or vehicle-control at 1 hpi, 6 hpi, 12 hpi, 18 hpi, 24 hpi, 30 hpi and 36 hpi. Supernatant from the infected cells was collected at 48 hpi and quantified by plaque assay^29,32^.

### Attachment assay

Confluent monolayers of Vero cells, in triplicates, were pre-incubated with 1 µg/ml Thiazovivin or 0.05% DMSO for 30 min followed by BPXV infection at MOI of 5 for 2 h at 4 °C. The cells were then washed 5 times with PBS and the cell lysates were prepared by rapid freeze–thaw method. The viral titers in cell lysates were quantified by plaque assay^29,32^.

### Entry assay

Confluent monolayers of Vero cells, in triplicates, were pre-chilled at 4 °C and infected with BPXV at MOI of 5 in Thiazovivin-free medium for 1.5 h at 4 °C, which allowed virus attachment to the host cells but restricted viral entry. Thereafter, the cells were washed with PBS and incubated with fresh DMEM containing 1 µg/ml Thiazovivin or 0.05% DMSO. To permit viral entry, cells were incubated at 37 °C for 1 h. Thereafter, cells were washed with PBS and grown in fresh DMEM without any inhibitor. Virus yield in the infected cell culture supernatants at 48 hpi was determined by plaque assay^29,32^.

### Virus release assay

Confluent monolayers of Vero cells, in triplicates, were infected with BPXV at MOI of 5 for 1 h. Thereafter, cells were washed with PBS and fresh DMEM was added. At 36 hpi, cells were washed 5 times with chilled PBS followed by addition of fresh DMEM containing 1.0 µg/ml Thiazovivin or equivalent volume of DMSO. Virus yield in the infected cell culture supernatant was quantified by plaque assay at 30 min and 2 h post-drug treatment^29,32^.

### qRT-PCR

The amount of viral DNA/mRNA(cDNA) in infected cells were measured by quantitative real-time PCR (qRT-PCR).Confluent monolayers of Vero cells, in triplicates, were infected with BPXV(MOI of 5) for 1 h followed by washing with PBS and addition of fresh DMEM. Thiazovivin (1 µg/ml) or DMSO (0.05%) were added at 6 hpi. Cells were scraped at 36 hpi to quantify the viral (*M* gene forward primer: 5′-AACACACATTATTCAGATACGTC-3′ and reverse primer: 5′-TTGTACGTCGCTCTTTGTTAG-3′) and house-keeping control gene (β-actin) by qRT-PCR as previously described^29^. The levels of viral DNA, expressed as threshold cycle (*Ct*) values, were normalized with β-actin housekeeping control gene. Relative fold-change in viral DNA copy number was determined by ΔΔ Ct method^56^.

### Effect of Thiazovivin on synthesis of viral proteins

Confluent monolayers of Vero cells were grown in 30 cm^2^ tissue culture dishes and infected with BPXV at MOI of 5. Inhibitors or vehicle-control were added at 6 hpi. Cells were scraped at 36 hpi to analyze the levels of viral-and house-keeping control proteins (β-actin) in Western blot analysis. Anti-BPXV serum was available at NCVTC Hisar and has been described elsewhere^29^.

### Selection of potential Thiazovivin-resistant virus variants

Vero cells were infected with BPXV at MOI of 0.1 in medium containing 0.05% DMSO or 0.2 µg/ml Thiazovivin. At 48–72 hpi, supernatant was collected from the virus infected cells [named passage 1 (P1)] and quantified by plaque assay. Fifty such sequential passages were carried out. The original virus stock (P0), P50-Thiazovivin and P50-Control viruses were used to infect Vero cells at an MOI of 0.1 with either 1.0 µg/ml Thiazovivin or 0.05% DMSO. At 48 hpi, viral titers in the infected cell culture supernatant were quantified by plaque assay^29,32^.

### Ligation-mediated Poly(A) tail length (LM-PAT)

LM-PAT assay was employed to measure the changes in poly(A) tail length by specifically targeting the oligo(dT) anchor at the end of the poly(A) tail as per the previously described method^57^. Briefly, at 9 h following BPXV infection, the cells were treated with Thiazovivin/Y27632- or DMSO in the presence of Actinomycin D. The RNA was isolated at 2 h and 4 h post-drug treatment. The poly(A) tail of mRNA was allowed to saturate with phosphorylated ATPs and then ligated with adaptor oligo(dT) primers (5′-GGCCACGCGTCGACTAGTACTTTTTTTTTTTTTTTT-3′) at 42 °C in the presence of T4 DNA ligase. The ligated mRNA was subjected to cDNA synthesis by using anchor primer 2 [specifically binds with ligated adaptor oligo(dT) primer; 5′-GGCCACGCGTCGACTAGTAC-3′]. Finally, the length of poly(A) tail was determined by PCR by using an anchor (primer 3; 5′-CUACUACUACUAGGCCACGCGTCGACTAGTAC-3′) and *M* gene-specific primer (5′-AGTATCAAAGATATAGATCACC-3′). Likewise, the length of poly(A) tail of endogenous/cellular mRNA was determined by PCR by using an anchor (primer 3; 5′-CUACUACUACUAGGCCACGCGTCGACTAGTAC-3′) and *β-actin*-specific primer (5′-CTTACCTGTACACTGACTTGA-3′).

### Measurement of mRNA stability

The stability of the mRNA was estimated as per the previously described method^58^. Briefly, Vero cells were infected with BPXV at MOI of 5. At 9 hpi, the cells were treated with Thiazovivin (1 µg/ml), Y27632 (1.5 µg/ml) or equivalent volume of DMSO in the presence of Actinomycin D (5 µg/ml). RNA was isolated at 0 h, 1 h, 2 h and 4 h following addition of the inhibitors and examined for the levels of BPXV *M* gene by qRT–PCR. The half‐lives of the targeted mRNA was calculated as described previously^58^.

### Chromatin immunoprecipitation (CHIP) assay

CHIP assay was carried out to evaluate the interaction of viral mRNA with a cellular poly(A) tail binding protein (PABP) by employing a previously described method^59^ along with some modifications. Briefly, Vero cells, in triplicates, were infected with BPXV at MOI of 5. At 9 hpi, when the RNA levels were expected to be at its peak, the cells were treated with ROCK1 inhibitors (Thiazovivin and Y27632) or DMSO. At 2 h and 4 h-post drug treatment, the cells were treated with 1% formaldehyde for 10 min to covalently cross-link interacting proteins and nucleic acid. Thereafter, the cross-linking reaction was stopped by addition of 125 mM glycine (final concentration) followed by washing the cells with ice-cold PBS. The cell lysates were prepared in immunoprecipitation (IP) buffer [150 mMNaCl, 50 mMTris-HCl (pH 7.5), 5 mM EDTA, 0.5% NP-40, 1% Triton X-100 plus protease and phosphatase inhibitor cocktail] and sonicated in a QsonicaSonicator Q500 (Qsonica, Newtown, CT, USA)(6 pulse of 15 s at amplitude of 40%). The cell lysates were then centrifuged for 10 min at 12,000 g. The clarified cell lysates were mixed with 10 units of RiboLock RNase Inhibitor (Thermo Scientific, USA) and then incubated with the PABP antibody (reactive antibody), phospho ERK antibody (non-reactive antibody) or equivalent volume of IP buffer (beads control) for 45 min at room temperature. Thereafter, 40 μl (5 ng/μl) of Protein A Sepharose® slurry, prepared as per the instruction of the manufacturer (Abcam, USA) was added into each reaction and incubated overnight at 4 °C on a rotary platform. The beads were then washed 5 times in the IP buffer (without protease inhibitors). To reverse the cross-linking, the complexes were then incubated with Proteinase K (20 mg/ml final concentration) at 56 °C for 40 min. Finally, the reaction mixtures were centrifuged at 12,000 g for 1 min and the supernatant was subjected to RNA isolation. The RNA was converted into cDNA and then the levels of BPXV *M* gene was quantified by qRT-PCR.

### Immunofluorescence assay

Vero cells were grown in chamber slides at ~ 20% confluency and infected with BPXV at MOI of 10 for 2 h, followed by washing with PBS and replacement with fresh medium. Thiazovivin was applied at 5 hpi. The intracellular localization of viral and/or cellular (p-MLC2) proteins in the virus-infected cells was detected by immunofluorescence assay at 15 hpi as described previously^51^.

### siRNA knockdown of ROCK1

In order to further confirm the role of ROCK1 in BPXV replication, as well as to avoid the possibilities that ROCK1 inhibitor may have some off-target effects in the host cells, ROCK1 was knockdown from the cells in a sequence dependent manner by using small interfering RNA (siRNA). Briefly, Vero cells were grown in 96 well plates. When the cells were at ∼75% confluency, 50 nmol and 100 nmol of ROCK1 (FlexiTube siRNA, Qiagen, Germany] or control siRNA (AllStars Neg Control siRNA, Qiagen, Germany] were transfected using Lipofectamine 3000 as per the instruction of manufacturer (Invitrogen, Carlsbad, USA). At 48 h post-transfection, cells were infected with BPXV at MOI of 1 and virus released in the infected cell culture supernatant at 48 hpi was quantified by plaque assay.

### *In ovo* antiviral efficacy of Thiazovivin against BPXV

#### Egg lethal dose 50 (LD_50_)

Specific pathogen free (SPF) embryonated chicken eggs were procured from Indovax Pvt Ltd, Hisar, India. LD_50_ of CGP57380 was determined by inoculating fivefold serial dilutions of Thiazovivin (concentration ranging from 50 to 0.0.02 µg/egg) or DMSO (vehicle-control), in 10 day old embryonated SPF eggs, in a total of 100 μl volumes via chorioallantoic membrane (CAM) route. Eggs were examined for the viability of the embryos up to five days post-inoculation to determine the LD_50_ by the Reed-Muench method^29,32^.

### ***In ovo*** antiviral efficacy (EC_50_)

SPF embryonated chicken eggs, in triplicates, were inoculated with fivefold serial dilutions (25 to 0.1 µg/egg) of Thiazovivin or equal volume of vehicle control via CAM route, followed by infection with BPXV at 100 EID_50_. On 5–7 dpi, eggs were examined for pock lesions and/or death of the embryos. EC_50_ was determined by the Reed-Muench method^29,32^.

### Statistical analysis

Statistical analysis was performed using the Graph Pad Prism 8 (GraphPad Software Inc., San Diego, CA, USA). The group difference was evaluated by student’s t test, error bars represent mean ± SD and the difference was statistically significant when *p < 0.05; **p < 0.01; or ***p < 0.001.

### Ethics approval

This study does not involve any experiment on humans or animals.

### Consent to participate

All authors agreed to participate.

## Supplementary Information

Supplementary Figures.

Supplementary Video 1.

Supplementary Video 2.

## Data Availability

Vero cell adapted BPXV was deposited to the repository at National Centre for Veterinary Type Cultures (NCVTC), Hisar with an Accession Number of VTCC-AVA90 and can be accessed at http://ncvtc.org.in/wp-content/uploads/2019/08/Viruses-availble-for-distribution.pdf. Its whole genome sequence is available in GenBank with an Accession Number of MW883892.1. All other data needed to evaluate the conclusions in this paper are present in the Supplementary Materials.
