# Supplementary material for: ROCK1/MLC2 inhibition induces decay of viral mRNA in BPXV infected cells
Source: Sci Rep. 2022 Oct 24;12:17811. doi: 10.1038/s41598-022-21610-9 (PMC9592580; doi:10.1038/s41598-022-21610-9)
Supplement: Supplementary file 1 — Supplementary Figures. [file 41598_2022_21610_MOESM1_ESM.pdf]

Supplementary Information

ROCK1/MLC2 inhibition induces decay of viral mRNA in BPXV infected cells

Ram Kumar[1,2,#], Yogesh Chander[1,3,#], Nitin Khandelwal[1], Assim Verma[1], Himanshu Nagori[1], Brij N. Shringi[2], Bhupendra N. Tripathi[1,$], Sanjay Barua[1], Naveen Kumar[1*]

Content:

Supplementary Figure 1. Determination of the cytotoxicity of ROCK1 inhibitors (MTT assay).

Supplementary Figure 2. siRNA knockdown of ROCK1.

Supplementary Figure 3. Virucidal activity of Thiazovivin and Y27632.

Supplementary Figure 4. Time-of-addition assay.

Supplementary Figure 5. ROCK inhibitor does not affect virus attachment, entry and budding.

Supplementary Figure 6. Kinetics of viral mRNA/DNA synthesis.

Supplementary Figure 7. Chemical structure of ROCK1 inhibitors.

Supplementary Figure 8. Full Image of Western blots and agarose gels.

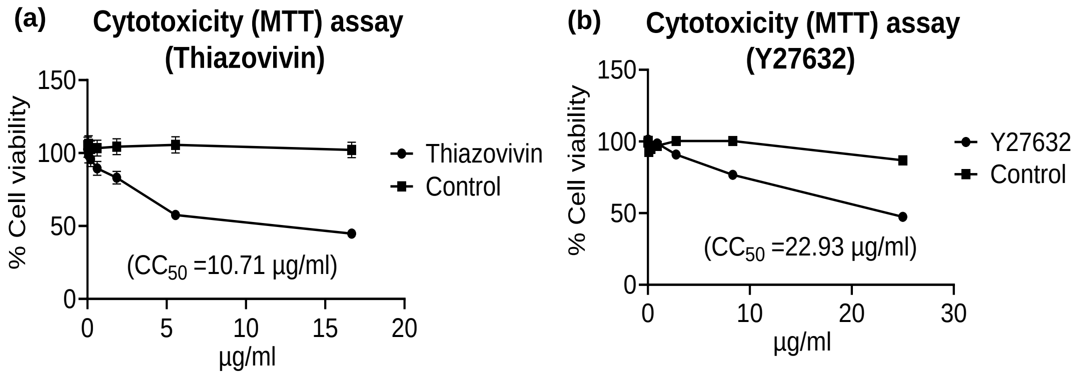

Supplementary Figure 1. ***Determination of the cytotoxicity of ROCK1 inhibitors (MTT assay).***

Indicated concentrations of ROCK1 inhibitors (Thiazovivin or Y27632) or equivalent volumes of vehicle control (DMSO) were incubated with cultured Vero cells (in triplicates) for 96 h and percentage of cell viability was measured by MTT assay. The percentage of cell viability at different concentration of Thiazovivin **(a)** and Y27632 **(b)** were shown.

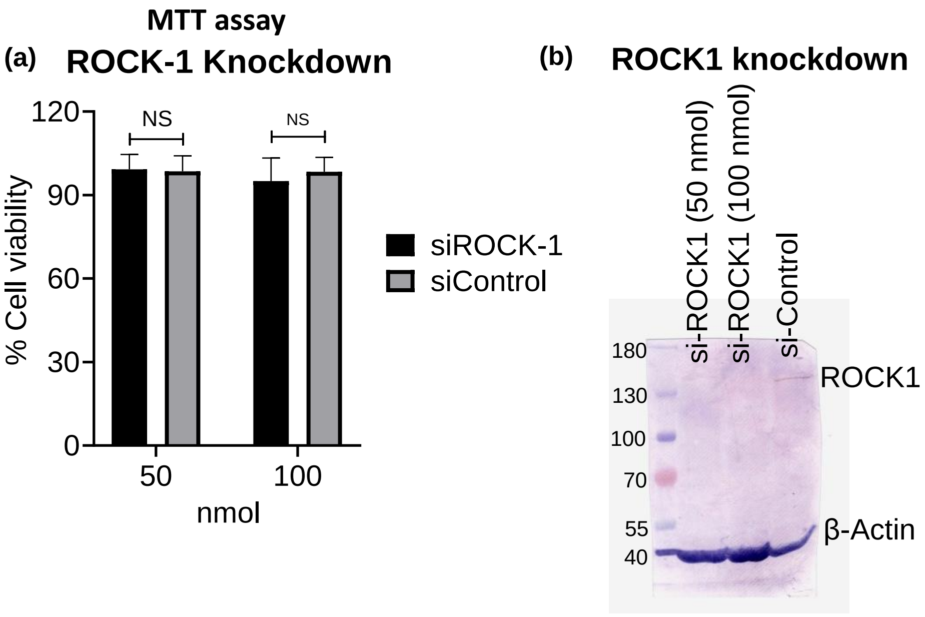

Supplementary Figure 2. ***siRNA knockdown of ROCK1.***
(a) Determination of the cytotoxicity of siROCK1 (MTT assay). Indicated concentrations of siROCK1 or siControl were transfected, in triplicates, in cultured Vero cells for 72 h and percentage of the cell viability was measured by MTT assay. The percentage of the cell viability at indicated concentrations of siROCK1 is shown. **(b) Evaluation on siRNA knockdown efficiency.** Vero cells were transfected with ROCK1 (50 nmol and 100 nmol) - or negative control (100 nmol) siRNAs. The cell lysates were prepared at 48 hpi. The levels of ROCK1 and β-Actin house-keeping control protein were determined by Western blot analysis.

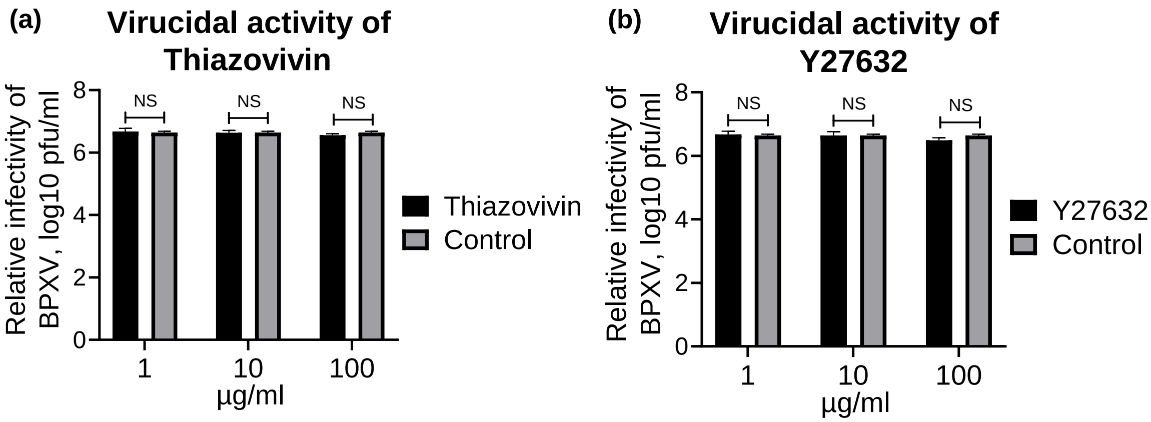

Supplementary Figure 3. ***Virucidal activity of Thiazovivin and Y27632.*** Indicated concentrations of the Thiazovivin and Y27632 or equivalent volumes of vehicle control were mixed with the virus (n=3) and incubated for 90 min at 37°C after which virus was diluted (1/1000) and relative infectivity was determined by plaque assay. The relative infectivity of BPXV of Thiazovivin **(a)** and Y27632 **(b)** were shown. Pair-wise statistical comparisons were performed using Student's t test. (NS=Non-significant difference)

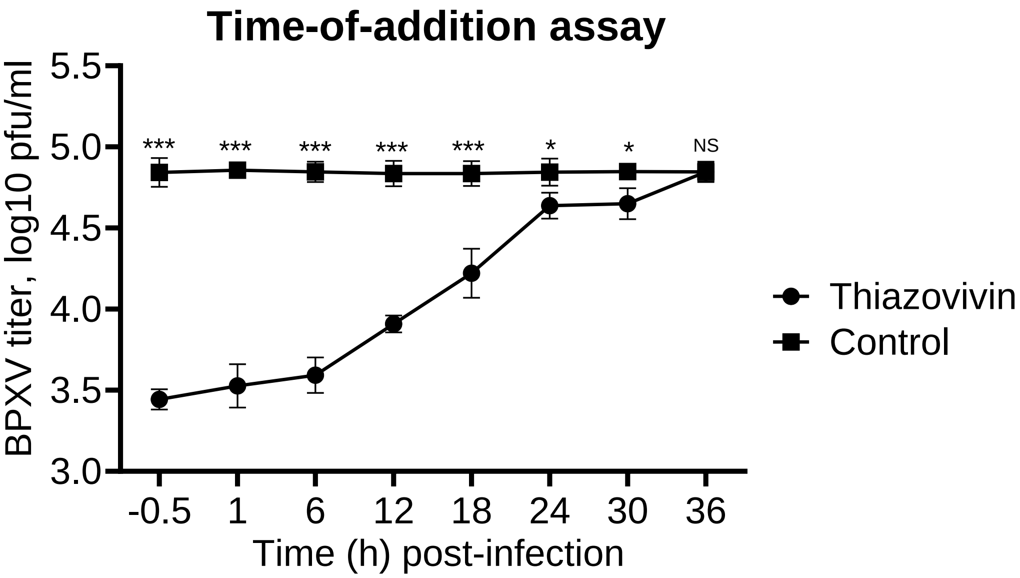

Supplementary Figure 4. *Time-of-addition assay*.
Pretreated (1 µg/ml of Thiazovivin) or untreated Vero cells were infected, in triplicates, with BPXV for 1 h. Inhibitor (Thiazovivin) or DMSO were periodically applied over the life cycle of BPXV at indicated time points. Supernatants were collected at 48 hpi and virus was quantified by plaque assay. . Pair-wise statistical comparisons were performed using Student's t-test (*** = P<0.001, * = P<0.05, NS=Non-significant difference).

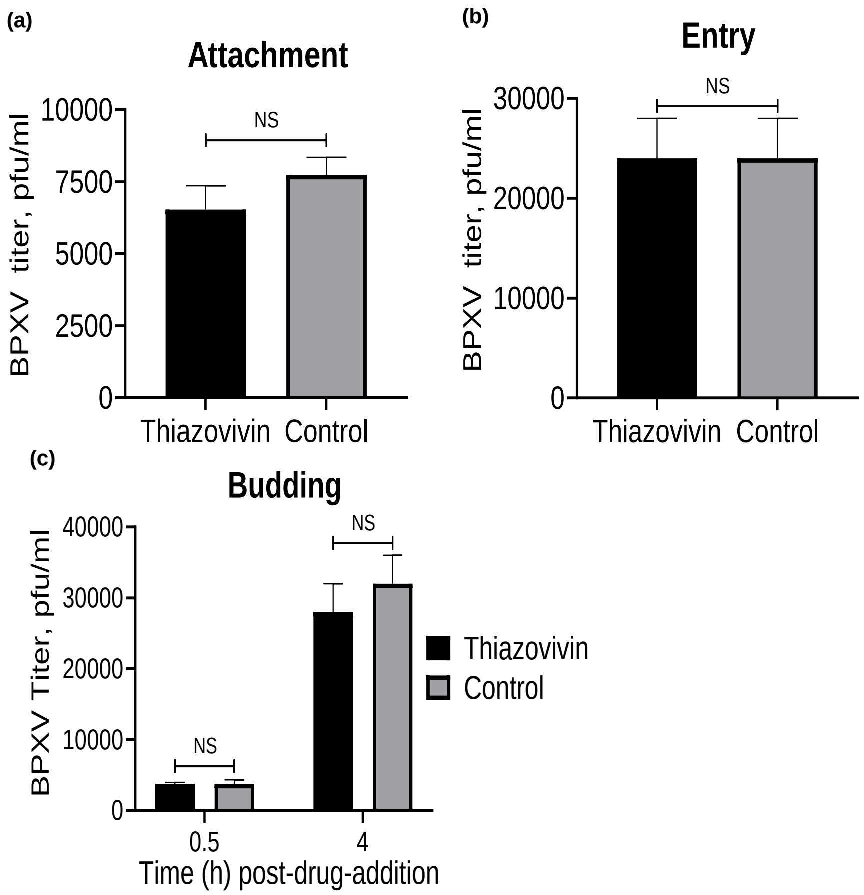

Supplementary Figure 5. ***ROCK inhibitor does not affect virus attachment, entry and budding.***

(a) Attachment assay. Vero cells were pre-incubated with Thiazovivin or DMSO for 30 min followed by infection with BPXV at 4°C for 1.5 h. Cells were then washed and cell lysates were prepared by rapid freeze-thaw method. Virus attached to the cells in the presence or absence of the inhibitor was quantified by plaque assay. ***(b) Entry assay.*** Confluent monolayers of Vero cells were infected with BPXV at 4°C for 1 h, followed by washing with ice-cold PBS. The attached virus was allowed to proceed for entry at 37°C in the presence of Thiazovivin or vehicle control for 1 h followed by washing and addition of fresh medium. Virus released in the infected cell culture supernatant at 40 hpi was quantified by plaque assay. ***(c) Budding assay.*** Confluent monolayers of Vero cells were infected with BPXV (MOI of 5) for 1 h, followed by washing with PBS and addition of fresh medium without inhibitor. At 36 hpi, when virus presumably starts budding, cells were again washed and fresh medium having 1 µg/ml of Thiazovivin or equivalent volumes of DMSO were added. Virus particles released at 30 min and at 4 h post-drug-treatment were quantified by plaque assay. Error bars indicate SD. Pair-wise statistical comparisons were performed using Student's t-test (NS=Non-significant difference).

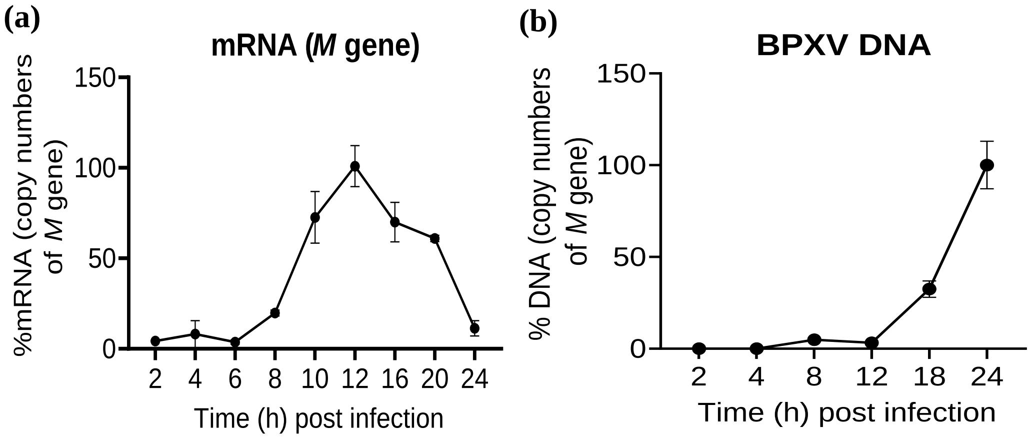

Supplementary Figure 6. *Kinetics of viral mRNA/DNA synthesis*.
(a) mRNA synthesis. The confluent monolayers of Vero cells, in triplicates were infected with BPXV at 5 MOI and scrapped at indicated time points to isolate the RNA. The DNAse1-treated RNA was subjected to quantify structural *M* (late Membrane protein encoding) genes by qRT-PCR. ***(b) DNA synthesis.*** The confluent monolayers of Vero cells, in triplicates were infected with BPXV at 5 MOI and scrapped at indicated time points to isolate the DNA. DNA was subjected to quantify BPXV *M* gene by qRT-PCR. Error bars indicate SD.

(a) Thiazovivin

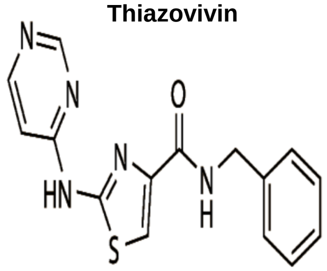

(b) Y27632

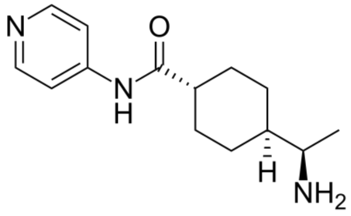

Supplementary Figure 7. ***Chemical structure of ROCK1 inhibitors*.**
(a) Thiazovivin [IUPAC name: N-benzyl-2-(pyrimidin-4-ylamino)-1,3-thiazole-4-carboxamide]. **(b).** Y27632 [IUPAC name: (R)-(+)-trans-4-(1-Aminoethyl)-N-(4-Pyridyl)cyclohexanecarboxamide dihydrochloride]

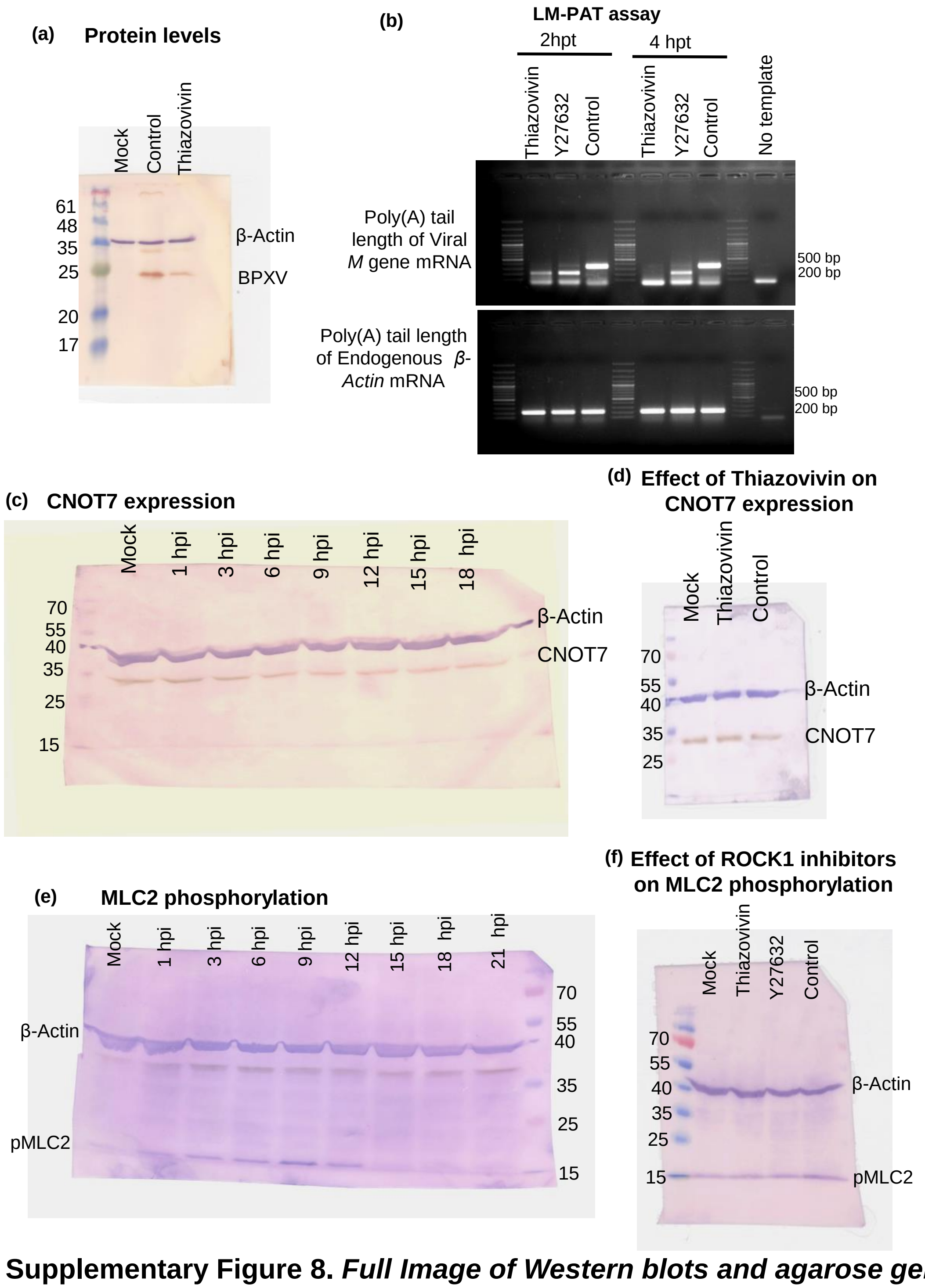

Supplementary Figure 8. ***Full Image of Western blots and agarose gels*****.**
(a) Full western blot corresponding to Figure 2ci**. (b)** Full agarose gel corresponding to Figure 3b**. (c)** Full western blot corresponding to Figure 3ci. **(d)** Full western blot corresponding to Figure 3di. **(e)** Full western blot corresponding to Figure 4bi. **(f)** Full western blot corresponding to Figure 5ai.
